# Synchronous Bilateral Breast Cancer and Thymoma: A Rare Case of Multiple Primary Malignancies

**DOI:** 10.1002/ccr3.72390

**Published:** 2026-04-03

**Authors:** Gholamreza Toogeh, Saeid Haji Aghajani, Seyyed Taher Seyyed Mahmoudi, Masoud Mortezazadeh, Hamidreza Zarei, Aysan Nozheh

**Affiliations:** ^1^ Thrombosis Hemostasis Research Center Tehran University of Medical Sciences Tehran Iran; ^2^ Division of Clinical Hematology Oncology and Bone Marrow Transplantation, Vali‐e‐Asr Hospital, Imam Khomeini Hospital Complex Tehran University of Medical Sciences Tehran Iran; ^3^ Tabriz University of Medical Sciences Tabriz Iran; ^4^ Department of Pathology, Cancer Institute, Imam Khomeini Hospital Complex Tehran University of Medical Sciences Tehran Iran

**Keywords:** adjuvant therapy, bilateral breast cancer, immunohistochemistry, synchronous malignancy, thymoma

## Abstract

Synchronous bilateral breast cancer and thymoma is rare, requiring vigilant surveillance and multidisciplinary management. This case highlights the importance of distinguishing multiple primary malignancies from metastases, emphasizing tailored multimodal therapy and long‐term follow‐up to address immune dysregulation and cancer predisposition.

## 1 Introduction

Thymoma, a rare thymic epithelial neoplasm, is strongly associated with secondary malignancies, with yet to be proven underlying mechanisms. Immune dysregulation and impaired tumor surveillance are hypothesized contributors, independent of radiotherapy or autoimmune conditions [1]. This cancer predisposition makes thymoma patients particularly challenging to manage, requiring vigilant long‐term monitoring. The synchronous occurrence of thymoma with other primary tumors presents unique diagnostic and therapeutic dilemmas [2]. This report illustrates these issues through a compelling case of a patient diagnosed with thymoma concurrently with another primary cancer.

## 2 Case History/Examination

A 46‐year‐old woman presented with a palpable mass in her right breast, discovered by self‐examination. She had no systemic symptoms, no prior surgeries, and an otherwise unremarkable medical history. Family history was negative for breast, ovarian, or thymic malignancies.

## 3 Differential Diagnosis, Investigations, and Treatment

Breast ultrasound revealed a well‐defined hypoechoic mass (14 × 12 mm) at 12 o'clock in the right breast. In the left breast, one heteroechoic lesion with irregular borders (11 × 7 mm) in the upper‐outer quadrant (2 o'clock) and one hypoechoic lesion with indistinct margins (9 × 7 mm) between 2 and 3 o'clock, approximately 4 mm from the skin surface, were identified. Mammography of the right breast showed calcified foci with probable polymorphic morphology in the upper‐outer quadrant (UOQ), an ill‐defined mass with marginal spiculation in the upper central region, focal asymmetric density in the upper inner quadrant (UIQ) and inferior aspect, enhanced parenchymal density in the central outer zone, subtle thickening of the periareolar skin, and bilateral retroareolar ductal prominence/density. (Mammography findings for the left breast were also performed but are omitted here.) Core needle biopsy results were as follows:

- Right breast (12 o'clock): ductal carcinoma in situ (DCIS) with solid and cribriform architecture, nuclear grade 2, focal necrosis, macrocalcifications. Immunohistochemistry: ER positive (30%–40%), Ki‐67 ~ 20%, p63 positive in the myoepithelial layer.
- Left breast: invasive breast carcinoma of no special type (NST), histologic grade 1/3 (modified Bloom–Richardson 4/9), mitotic count 1, nuclear pleomorphism 2. Adjacent DCIS (solid pattern, nuclear grade 2, no necrosis). ER strong (90%–100%), PR positive (70%–80%), HER2 negative, Ki‐67 low (~6%).
- Additional biopsy of a right UOQ lesion revealed pseudoangiomatous stromal hyperplasia (PASH), with no evidence of malignancy or atypia (Figure S1).

In accordance with NCCN guidelines for clinical stage I/II breast cancer, and given the absence of pulmonary symptoms or clinically positive axillary nodes, a preoperative chest CT was not performed. The mediastinal mass was subsequently identified on the restaging CT performed to monitor chemotherapy response. Following the histological confirmation of synchronous bilateral breast cancer in a young patient, genetic counseling and germline testing were strongly recommended to help guide surgical and systemic management. However, the patient deferred testing due to financial constraints.

Although breast‐conserving surgery was discussed, a bilateral mastectomy was elected. The medical rationale included the multicentricity of the left‐sided disease (lesions in the UOQ and lower quadrant) and the extensive DCIS component in the right breast, which posed a high risk for positive margins and recurrence. Additionally, alongside the oncological indications, a bilateral approach was chosen to alleviate severe psychological distress and align with the patient's strong preference for maximal risk reduction. Consequently, the patient underwent bilateral modified radical mastectomy with pectoral muscle preservation. Left breast: Two separate lesions:

1. Primary tumor in UOQ, 2.3 cm, classic invasive lobular carcinoma, grade 2/3 (Bloom–Richardson 6/9): tubule/gland formation 3, nuclear pleomorphism 2, mitotic rate 1.
2. Secondary lesion (1.7 cm) inferolateral: mixed histology (60% invasive breast carcinoma NST + 40% invasive lobular), grade 1/3 (score 4/9): differentiation 1, pleomorphism 2, mitoses 1.

**FIGURE 1:**
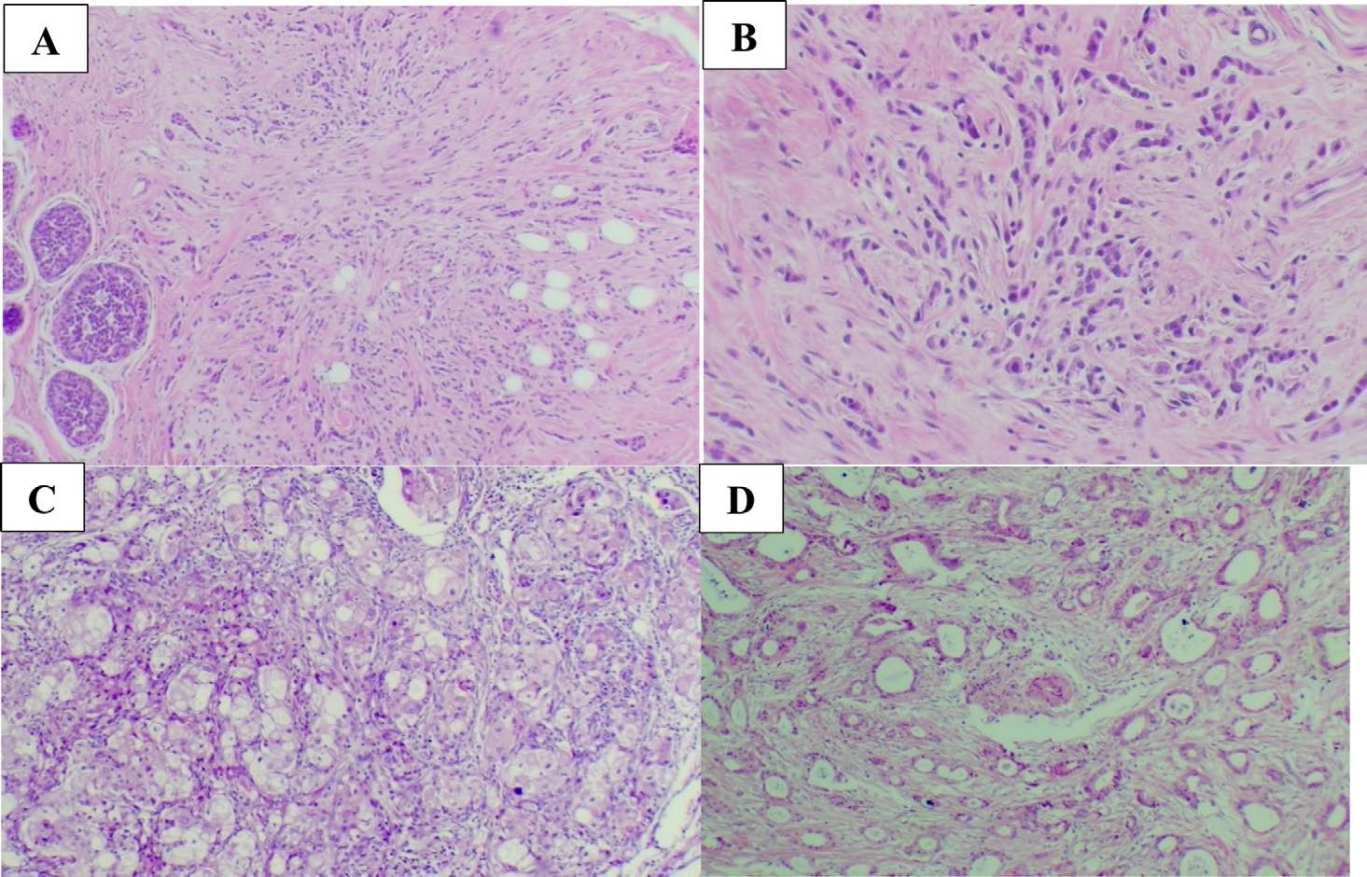
Histological sections stained with hematoxylin and eosin (H&E); (A, B) (at magnification x100 and x400, respectively): Invasive lobular carcinoma component. (C, D) (at magnification x100): Invasive ductal carcinoma component.

Both had in situ components: DCIS (solid & cribriform, nuclear grade 2, no necrosis) and lobular carcinoma in situ. There was lymphovascular invasion, but no dermal involvement or microcalcifications. Final pathologic stage: pT2 pN0 (AJCC 8th edition) (Figure 1).

Right breast: Invasive breast carcinoma of no special type (NST) with apocrine differentiation, 1.5 cm (at 12 o'clock), grade 2 (Bloom–Richardson 7/9): differentiation 3, pleomorphism 3, mitotic rate 1. Associated extensive DCIS (solid + cribriform), high nuclear grade (grade 3), focal necrosis. No LCIS. No lymphovascular or dermal involvement. Pathologic stage: pT1c pN0 (AJCC 8th).

Despite the hormone receptor positivity, the multidisciplinary team (MDT) recommended adjuvant chemotherapy prior to endocrine therapy. This decision was based on high‐risk features, including the patient's premenopausal status (age 46), the bilateral nature of the disease, and specifically the biology of the right‐sided tumor, which presented with High Nuclear Grade 3 and elevated Ki‐67, indicative of a Luminal B‐like subtype necessitating cytotoxic intervention. Genomic profiling (e.g., Oncotype DX) was unavailable due to resource limitations. Post‐surgery, the patient was started on adjuvant chemotherapy: 4 cycles of AC (doxorubicin/Adriamycin + cyclophosphamide) followed by 4 cycles of paclitaxel.

**FIGURE 2:**
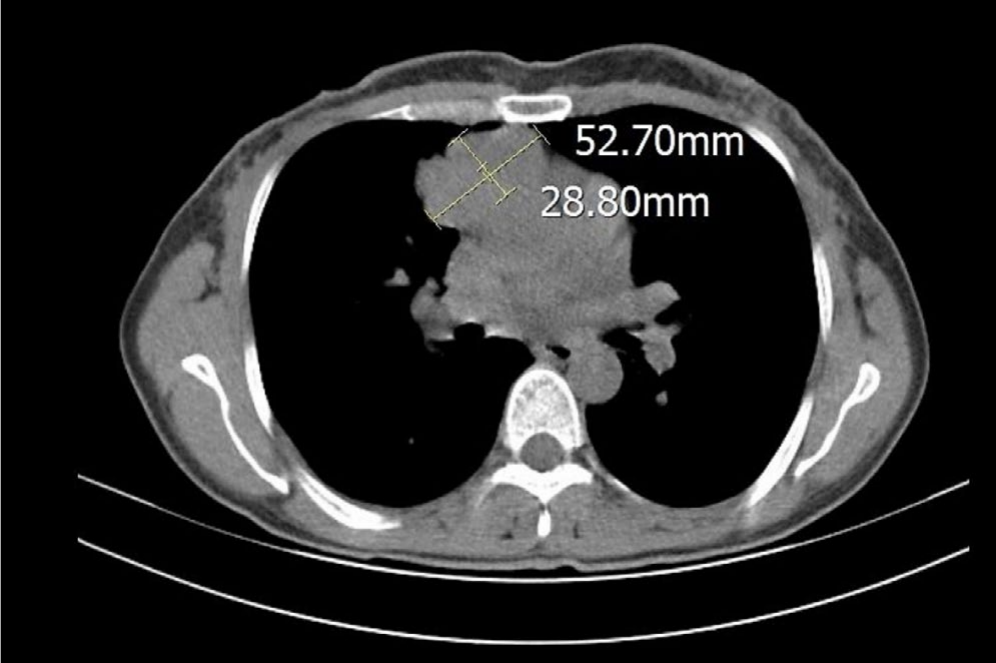
Contrast‐enhanced chest computed tomography (CT) (axial view) demonstrating a heterogeneous anterior mediastinal mass measuring 52 × 28 mm.

Two months after mastectomy, imaging detected an anterior mediastinal mass. High‐resolution CT showed a roughly 52 × 28 mm mass in the anterior mediastinum (Figure 2). CT‐guided core needle biopsy with immunohistochemistry revealed epithelioid tumor cells positive for PAX‐8, background lymphocytes positive for CD3 and TdT. Morphology and IHC were consistent with thymoma; metastasis from breast cancer was ruled out (Figure S2).

**FIGURE 3:**
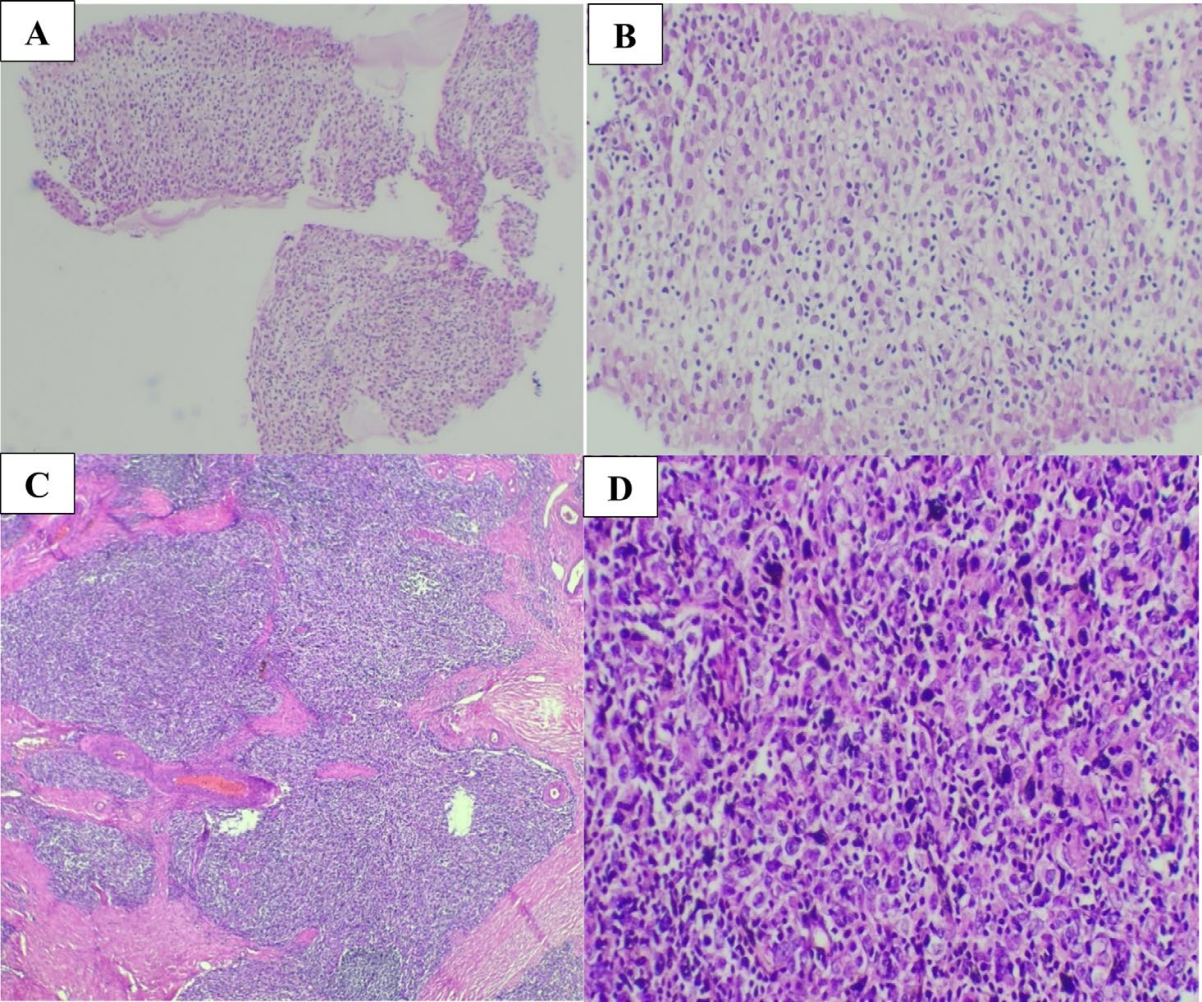
Histological sections stained with hematoxylin and eosin (H&E); (A, B) (at magnification x100 and x400, respectively): Mediastinal mass biopsy in favor of thymoma type B2. (C, D) (at magnification x40 and x400, respectively): Resection of thymus shows invasive thymoma type B2 (50%) and B3 (50%).

After completing breast cancer chemotherapy, a MDT decision led to surgical resection of the mediastinal mass. The patient underwent thymectomy with wedge resection of the right upper lobe. Pathology showed an invasive thymoma measuring 6 cm, comprising roughly equal portions of WHO type B2 and B3 thymoma. Lung margin was clear; no lymphovascular invasion. Classified as stage III by the modified Masaoka (Masaoka–Koga) staging system; pathologic stage pT3N0M0 (Figure 3). Adjuvant radiotherapy was administered (25 sessions).

## 4 Outcome and Follow‐Up

Endocrine therapy comprising letrozole and a GnRH agonist was initiated two weeks after the completion of chemotherapy. Surveillance included physical examinations every six months with special attention to surgical sites, routine biochemical monitoring for the toxicity of hormonal therapy, and tumor marker checks. For thymoma, contrast‐enhanced thoracic CT scans were performed every six months. At 3‐year follow‐up, the patient remains in good general health with no evidence of recurrence of either breast cancer or thymoma.

## 5 Discussion

This case highlights the rare coexistence of synchronous bilateral breast carcinoma and thymoma, underscoring the diagnostic and therapeutic challenges of managing multiple primary malignancies. Thymoma remains a rare cancer without a clear etiology, and despite limited articles about its epidemiology, it represents only 0.2%–1.5% of all malignancies [3]. Thymoma, regarded as the most frequent primary tumor of the thymus, arises from thymic epithelial cells that regulate T‐cell maturation. Characterized by a dense infiltrate of T lymphocytes, this condition can release aberrant cells into circulation, triggering various autoimmune manifestations, including myasthenia gravis (MG) (the most prevalent association), hematologic disorders, and connective tissue disease [4, 5].

Patients with thymoma exhibit a significantly elevated risk of secondary malignancies, with studies reporting a prevalence up to 18 times higher than the general population, manifesting both prior to and following the diagnosis of thymoma [6]. Common secondary cancers include lymphoma, breast, lung, prostate, and colorectal carcinomas, though the risk profile varies across studies [6, 7]. Other studies have also highlighted colorectal and thyroid cancer as the most common secondary primary malignancies to thymoma [8]. The simultaneous occurrence of these two distinct cancers is exceedingly uncommon, and only isolated case reports have been published.

The mechanisms by which thymoma may evoke extrathymic neoplasms remain poorly understood, but several hypotheses have been proposed. One theory posits that thymoma‐induced immune dysregulation, characterized by aberrant T‐cell selection, may impair immune surveillance, predisposing patients to oncogenesis [9]. This is supported by studies showing elevated cancer risk independent of thymectomy, suggesting an intrinsic thymoma‐related effect rather than a surgical consequence [10, 11]. The link between MG and increased extrathymic malignancy risk in thymoma patients is debated. Recent evidence suggests MG's immunological mechanisms do not elevate cancer risk, which is an intrinsic thymoma trait, independent of autoimmune conditions like MG and non‐specific to cancer type [12]. Radiation therapy, often used post‐thymectomy, has been investigated as a potential cause of secondary malignancies. Pan et al. indicated that the association between thymoma and secondary malignancies could not be related to radiation therapy [8], as in our patient who underwent radiation therapy after thymectomy. Also, the idea that the biological behavior of thymoma did not correlate with extrathymic malignancies was mentioned in this study. Also, the relation of diagnosed sarcoma and thymoma was investigated in a case report done by Balbino et al. [7] with no genetic predisposition, highlighting the fact that thymoma has a predisposition to the development of further neoplasms, as can occur with breast cancer.

To strictly categorize this case, we utilized the Warren and Gates criteria as cited by Mardani et al. [13], which mandate that each tumor must present a definite picture of malignancy, be distinct, and exclude the possibility that one is a metastasis of the other [13]. Furthermore, while metachronous tumors are separated by more than six months, our case falls within the definition of synchronous multiple primary malignant neoplasms (SMPMNs). As noted by Jia et al., SMPMNs are significantly rarer than their metachronous counterparts, accounting for a minority of multiple primary cancers and presenting a unique diagnostic burden [14]. The coexistence of thymoma with bilateral breast cancer, specifically, is an exceptionally rare subset of this category. Although the thymoma was histologically confirmed two months after the mastectomy, this falls well within the widely accepted surveillance definition (e.g., SEER criteria) of synchronous malignancies, defined as distinct primary tumors diagnosed within six months of one another.

The synchronous occurrence of both thymoma and breast cancer is rare. To our knowledge, there are few case reports indicating this incidence. First is a case reported by Athansiou et al. [1], presenting a 56‐year‐old female presented with invasive lobular breast carcinoma, incidentally found to have a synchronous thymoma during routine staging evaluations. The patient underwent concurrent surgical resection of both primary tumors. Comparative analysis with recent literature reveals striking parallels and key differences. De Placido et al. reported two cases of synchronous thymic and breast malignancies, including a 60‐year‐old female with invasive breast cancer (NST) and a synchronous B2/B3 thymoma—histologically identical to our patient's thymic component [15]. However, unlike their case, where the thymoma was Stage I (pT1a), our patient presented with a locally advanced (Stage III) thymoma, necessitating a more aggressive consolidation with radiotherapy. Furthermore, while Mardani et al. described a B3 thymoma in a patient with multiple malignancies, their case was metachronous and involved a triad of rectal, breast, and thymic neoplasms over 15 years [13]. The synchronous presentation in our case posed a more acute therapeutic challenge than the sequential management described in metachronous reports.

A primary challenge in this case was the incidental discovery of the thymoma. Current NCCN guidelines for early‐stage breast cancer (cT1‐2, N0) do not recommend routine systemic staging with chest CT in asymptomatic patients to avoid unnecessary radiation and false positives. Consequently, the thymoma was only detected post‐mastectomy. This highlights a diagnostic blind spot; however, the synchronous management remained feasible. The therapeutic challenge in synchronous malignancies lies in the lack of standardized guidelines. As reviewed by De Luca et al., while metachronous tumors allow for sequential standard‐of‐care treatments, synchronous presentations force a trade‐off between targeting the most aggressive malignancy and managing the comorbidities of multiple therapies [16]. Our approach aligns with the strategy proposed by Jia et al., who advocate for independent staging of each tumor immediately upon diagnosis to guide the surgical sequence [14]. In our case, the ‘challenge’ was the incidental discovery of the thymoma post‐mastectomy. Had the thymoma been identified preoperatively, a simultaneous resection might have been considered; however, the staged approach (chemotherapy followed by thymectomy) ultimately allowed for adequate recovery and uncompromised dosing of adjuvant breast regimens.

In our case, the patient demonstrated bilateral breast cancer with histological heterogeneity, including invasive carcinoma NST and lobular carcinoma components. Hormone receptor positivity and HER2 negativity in both breasts guided the decision toward endocrine therapy in addition to cytotoxic chemotherapy. This aligns with current guidelines recommending individualized multimodal therapy for synchronous bilateral breast cancer. The incidental discovery of an anterior mediastinal mass during breast cancer follow‐up highlights the importance of vigilant surveillance in patients with complex malignancies. The histological classification of thymoma (type B2/B3, invasive, Masaoka stage III) carries a significant risk of recurrence, justifying the combined approach of surgical resection and adjuvant radiotherapy. The MDT approach was critical in sequencing therapies to ensure both cancers were addressed effectively without compromising systemic control.

From a clinical standpoint, several lessons can be drawn from this case. First, synchronous tumors should always be considered in patients with atypical or incidental imaging findings, even when a known malignancy is present. When a mediastinal mass is detected following a breast cancer diagnosis, it is essential to differentiate it from metastatic disease. Metastatic involvement of the thymus is exceedingly rare among solid malignancies, including breast cancer [17]. Several mechanisms account for this rarity: the thymus constitutes a highly immunologically active microenvironment hostile to tumor cell colonization, the blood–thymus barrier prevents hematogenous extravasation, and the thymus lacks a permissive pre‐metastatic niche. Therefore, accurate histopathological distinction between a breast cancer metastasis and a second primary thymic tumor (e.g., thymoma or lymphoma) via core needle biopsy is essential, as therapeutic implications differ substantially. Third, the coordination of systemic therapy for breast cancer with local therapy for thymoma exemplifies the importance of MDT‐based decision‐making in complex oncologic care. Long‐term follow‐up is essential in such patients, given the propensity of both breast cancer and thymoma for late recurrence. Our patient's favorable outcome at three years underscores the effectiveness of comprehensive multimodal treatment. However, ongoing surveillance remains warranted to detect potential recurrences or additional secondary malignancies, given the underlying immune dysregulation associated with thymoma. This case is distinct not only due to the synchronous presentation but also the histological heterogeneity (invasive lobular carcinoma, invasive breast carcinoma NST, and thymoma type B2/B3) in a single patient. It underscores that immune dysregulation in thymoma patients may manifest as susceptibility to common malignancies like breast cancer, even in the absence of overt autoimmune disease. In conclusion, this case contributes to the limited body of literature on synchronous thymoma and bilateral breast cancer. It reinforces the role of immune dysfunction in cancer predisposition, highlights the diagnostic pitfalls in distinguishing metastatic disease from second primaries, and demonstrates the value of MDT‐guided, patient‐tailored management. Reporting such cases is crucial to improving our understanding of the clinical course and optimizing treatment strategies for patients with rare synchronous malignancies.

## Author Contributions

**Gholamreza Toogeh:** conceptualization, project administration, supervision, visualization, writing – review and editing. **Saeid Haji Aghajani:** conceptualization, project administration, supervision. **Seyyed Taher Seyyed Mahmoudi:** supervision, writing – original draft, writing – review and editing. **Masoud Mortezazadeh:** project administration, supervision, validation, visualization. **Hamidreza Zarei:** conceptualization, supervision, validation. **Aysan Nozheh:** formal analysis, investigation, methodology, resources, supervision.

## Funding

The authors have nothing to report.

## Ethics Statement

In this study, no additional costs were imposed on the patient. We maintained the patient's privacy, and written consent was obtained.

## Consent

A written informed consent was obtained from the patient.

## Conflicts of Interest

The authors declare no conflicts of interest.

## Supporting information


**Figure S1:** Immunohistochemistry staining of breast mass at magnification x400; (A) ER, strong positive in 90%–100% of tumor cells, (B) PR, strong positive in 70%–80% of tumor cells, (C) HER‐2, negative score 0, (D) Ki67, average 20%.
**Figure S2:** Immunohistochemistry staining of mediastinal mass at magnification x400; (A) PAX8, strongly positive in epithelioid cells, (B) TTF1, negative, (C) TdT, positive in background lymphocytes, (D) CD3, positive in background lymphocytes, (E) ER, negative.

## Data Availability

The data that support the findings of this study are available from the corresponding author [S.T.S.M.], upon reasonable request.
