# Supplementary material for: Synchronous Bilateral Breast Cancer and Thymoma: A Rare Case of Multiple Primary Malignancies
Source: Clin Case Rep. 2026 Apr 3;14(4):e72390. doi: 10.1002/ccr3.72390 (PMC13052234; doi:10.1002/ccr3.72390)
Supplement: Supplementary file 1 — Figure S1: Immunohistochemistry staining of breast mass at magnification x400; (A) ER, strong positive in 90%–100% of tumor cells, (B) PR, strong positive in 70%–80% of tumor cells, (C) HER‐2, negative score 0, (D) Ki67, average 20%. Figure S2: Immunohistochemistry staining of mediastinal mass at magnification x400; (A) PAX8, strongly positive in epithelioid cells, (B) TTF1, negative, (C) TdT, positive in background lymphocytes, (D) CD3, positive in background lymphocytes, (E) ER, negative. [file CCR3-14-e72390-s001.docx]

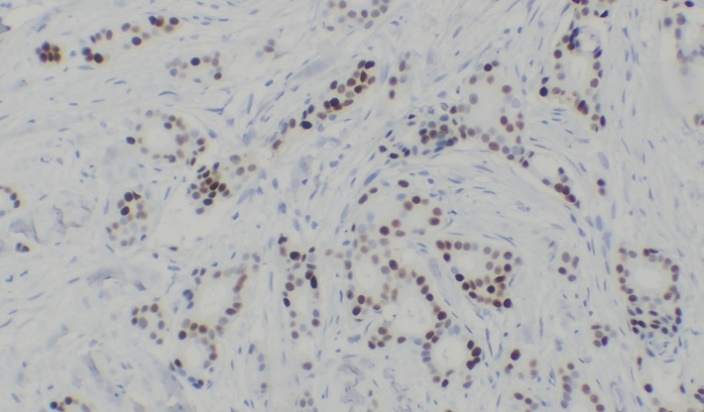

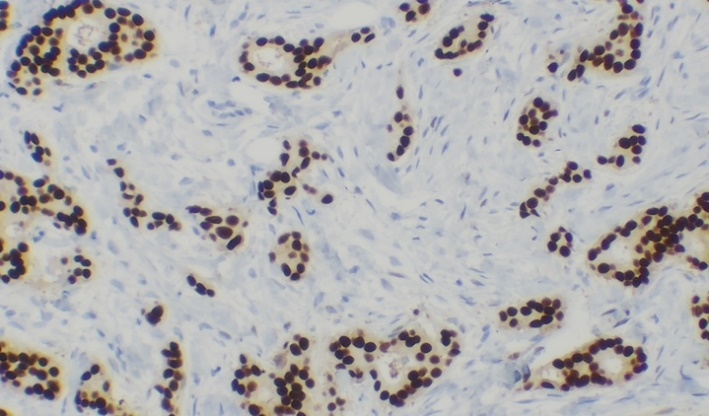


**B**

**A**


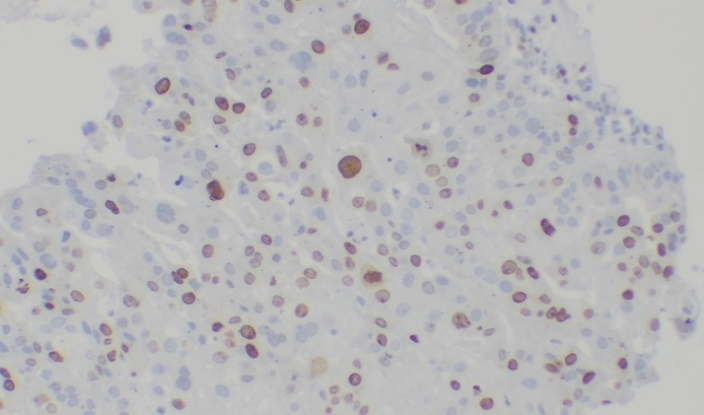

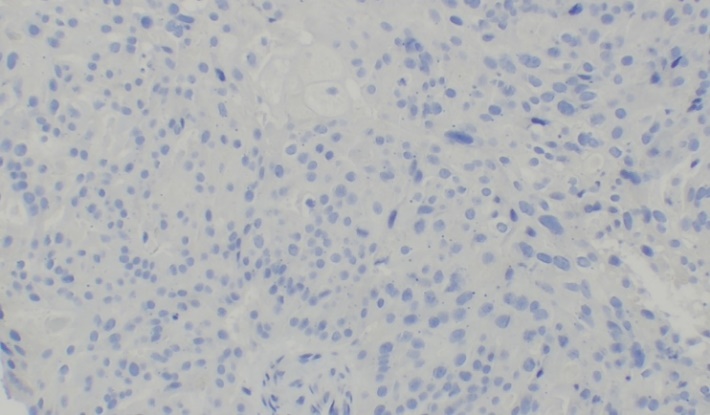


**D**

**C**

**Figure S1)** Immunohistochemistry staining of breast mass at magnification x400; A: ER, strong positive in 90-100% of tumor cells, B: PR, strong positive in 70-80% of tumor cells, C: HER-2, negative score 0, D: Ki67, average 20%.


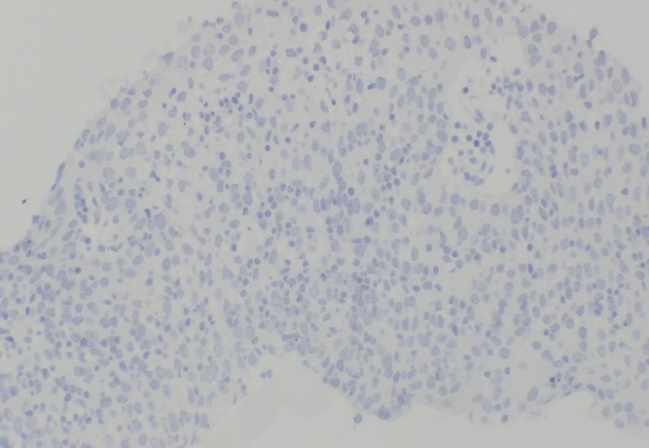

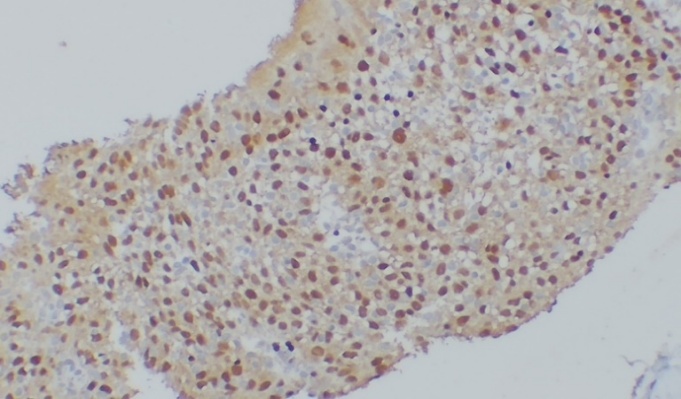


**B**

**A**


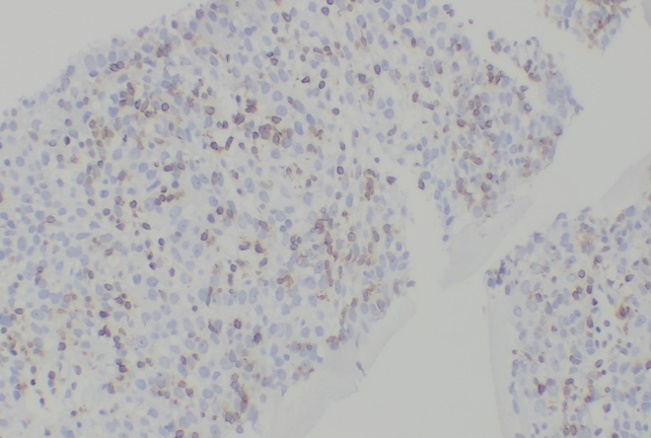

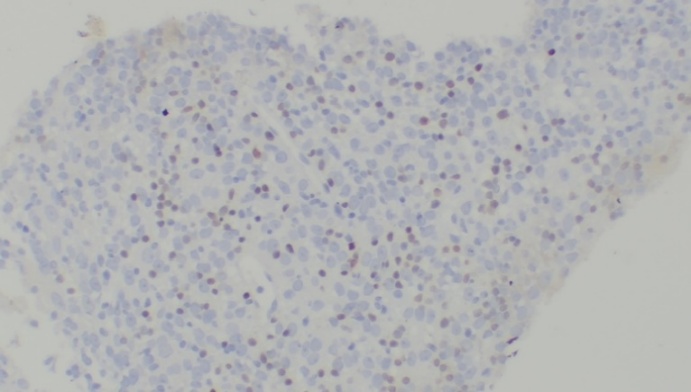


**D**

**C**


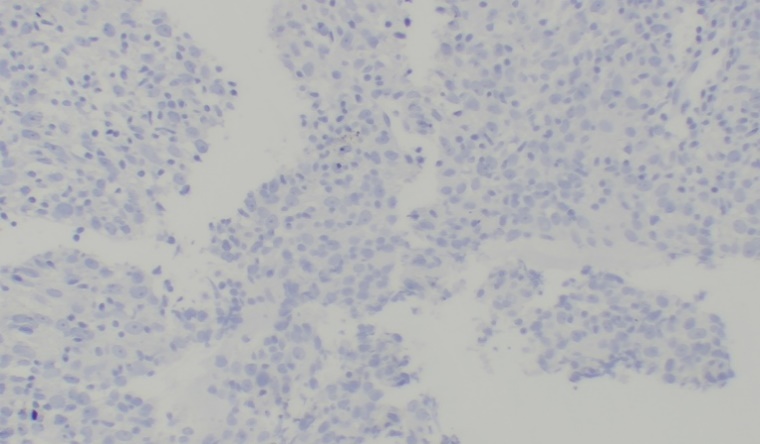


**E**

**Figure S2)** Immunohistochemistry staining of mediastinal mass at magnification x400; A: PAX8, strongly positive in epithelioid cells, B: TTF1, negative, C: TdT, positive in background lymphocytes, D: CD3, positive in background lymphocytes, E: ER, negative.
